# Endovascular foreign body removal of a MynxGrip polyethylene glycol (PEG) sealant embolus using a cerebral stent retriever

**DOI:** 10.1186/s42155-020-00197-0

**Published:** 2021-03-01

**Authors:** Mohammed Shamseldin, Hendrik Bergert, Axel Neumeister, Ralf Puls

**Affiliations:** Institute of diagnostic and interventional radiology, Helios Klikum, Erfurt, Germany

**Keywords:** MynxGrip, Vascular closure device, Stent retriever, pRESET, Foreign body removal

## Abstract

**Background:**

This is a rare case of removing an intra-arterial foreign body represented by MynxGrip polyethylene glycol (PEG) sealant as a rare complication of using the MynxGrip™ Vascular Closure Device (AccessClosure, Inc., Mountain View, CA) using a pRESET stent retriever (Phenox, Bochum, Germany) which is utilized mainly for treatment of endovascular stroke.

**Case presentation:**

A 60-year-old female patient suffering from intermittent claudication in the right lower limb (stage IIb according to Fontaine) due to a peripheral arterial occlusive disease was presented for an elective revascularization using balloon angioplasty of a short chronic occlusion of the right superficial femoral artery. After a successful revascularization of the right superficial femoral artery using a retrograde femoral access from the left common femoral artery, the patient suffered from an acute limb ischemia in the left foot with distal popliteal embolization with involvement of BTK (below the knee) trifurcation. This is believed to be due to an intra-arterial foreign body embolism of MynxGrip polyethylene glycol sealant as a rare complication of using the MynxGrip™ Vascular Closure Device.

**Conclusions:**

Stent retrievers have been used previously in removing dislocated coils especially in the cerebral vessels. This case report however proves a high efficacy and safety of using stent retrievers in removing different and rather unusual intra-arterial foreign bodies such as MynxGrip polyethylene glycol sealant.

## Introduction

The MynxGrip™ Vascular Closure Device (AccessClosure, Inc., Mountain View, CA) is nowadays a widely spread used and safe active closure device, which is the next generation of the previously known passive closure device namely the Mynx™ Vascular Closure Device (Thai and Weinstock 2012; Fields et al. 2010). The main development of the device was adding the proprietary Grip technology to the end of the original Mynx sealant. The Grip Tip and the Mynx sealant together form the MynxGrip sealant, which reacts to the pH level and the body temperature once deployed below the level of the skin and on the outer vessel wall. This reaction causes the sealant to soften and swell quite rapidly and thereby fully and actively closing the arteriotomy site together with the tissue tract (Thai and Weinstock 2012). The whole deployment process is complete within about 3 min.

Based on our experience using over a thousand MynxGrip devices, the overall complications using MynxGrip are extremely rare (< 1%). One of those quite rare but rather severe complications was accidentally pushing the Mynx sealant into the artery during application of the device resulting in a distal foreign body embolization with signs and symptoms of an acute limb ischemia (Fields et al. 2010; Gupta et al. 2012). This complication occurred twice in our hospital and studies showing the rates of such complications in other centers are to our knowledge nonexistent.

Based on our experience in using pRESET stent retrieves (Phenox, Bochum, Germany) in removing dislocated coils, we were able to successfully use the stent retriever in removing the dislodged Mynx sealant from the distal popliteal artery and the BTK trifurcation.

## Case report

A 60-year-old female patient suffering from a peripheral arterial occlusive disease stage IIb on the right side according to Fontaine was presented for an elective revascularization using balloon angioplasty due to a short occlusion of the distal superficial femoral artery. A retrograde femoral access from the left common femoral artery was decided and the intervention took place with a technical success. Later that day, the patient showed signs and symptoms of an acute limb ischemia in the left foot. Doppler, Duplex and CTA confirmed an acute occlusion of the distal left popliteal artery as well as BTK trifurcation. Due to our previous experience with a similar case, which our surgeons treated surgically after failure of endovascular mechanical thrombectomy using an aspiration catheter and was histologically confirmed to match the PEG sealant of a MynxGrip device, we immediately considered this a similar case of a MynxGrip PEG sealant embolization. The previous attempt to remove the PEG sealant probably did not work due to the consistency of the sealant, which is quite different to that of blood clots.

A retrograde femoral access from the right common femoral artery was decided with using a 60 cm long 7F Destination® Guiding Sheath (Terumo, Tokyo, Japan). For thrombectomy a SOFIA 6F aspiration catheter (Microvention Inc., Aliso Viejo, CA) and a pRESET stent retriever were used.

**Fig. 1:**
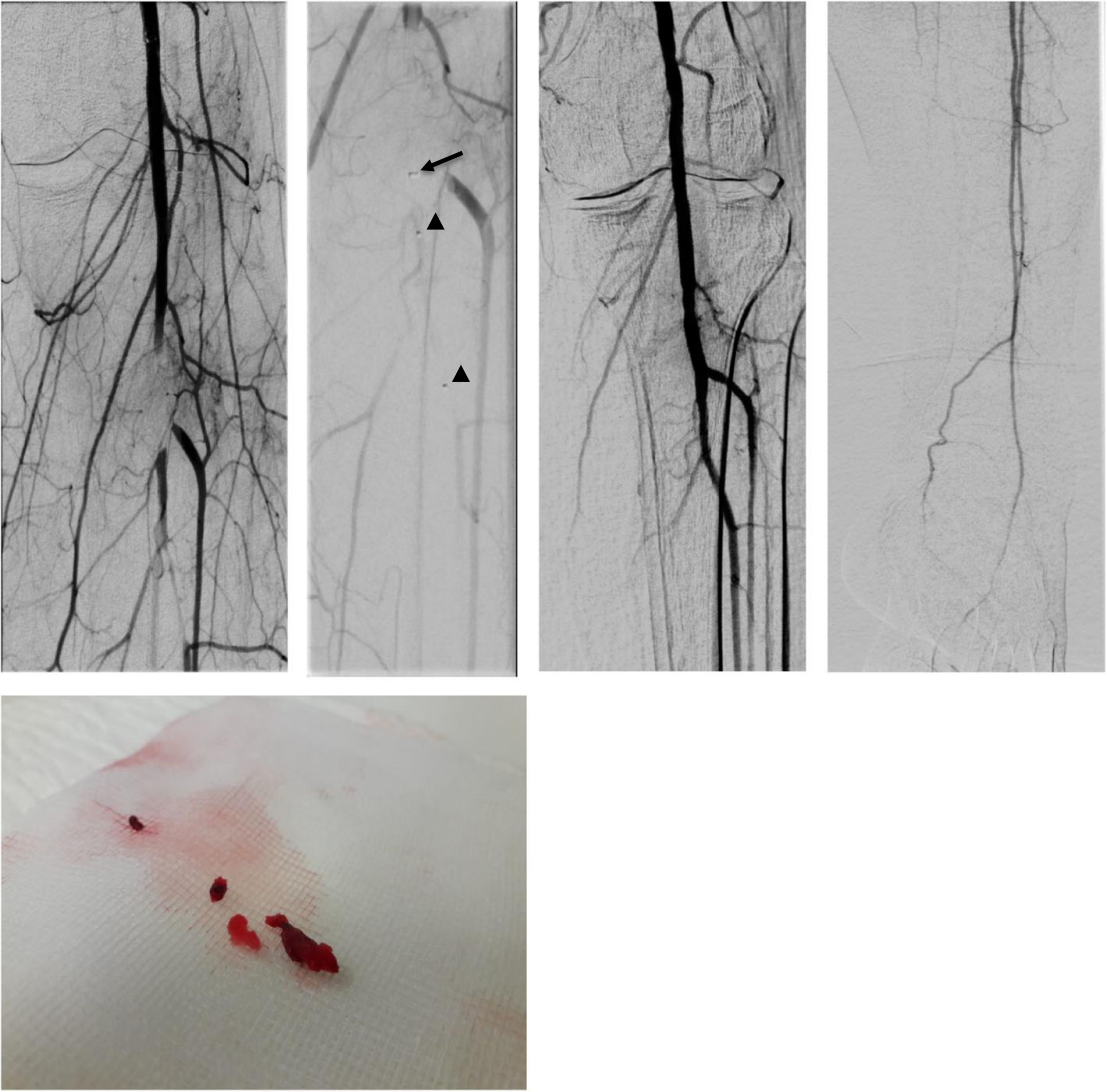
**a** acute foreign body embolisation caused by accidentaly pushing the Mynx sealant into the artery during application of the MynxGrip closure device **b** advancement of the SOFIA aspiration catheter (black arrow) to the proximal end of the foreign body and deploying the pRESET over the foreign body (arrow heads) **c** digital subtraction angiography documents regular flow after complete removal of the foreign body **d** final picture of the leg arteries showing absence of distal embolization of the foreign body with previously known chronic occlusion of the posterior tibial artery **e** removed foreign body (Mynx PEG Sealant)

We decided to use a pRESET stent retriever with a diameter of 6 mm (as the popliteal artery measurement during the intervention was 5 mm) and a length of 30 mm (which is enough to cover up the entire embolus length). The sheath was placed about 3 cm proximal to the foreign body, then the SOFIA aspiration catheter was placed at the proximal end of the foreign body (Fig. 1a) and the pRESET was advanced through a Rebar®-18 Micro Catheter (ev3, Minneapolis, USA) and deployed over the foreign body with the foreign body at the upper end of the deployed pRESET (Fig. 1b). The retrieval took place in two steps. The first step was pulling back the pRESET into the SOFIA catheter together with simultaneous manual aspiration over the SOFIA catheter. The second step was a successive manual aspiration over the sheath as soon as the SOFIA catheter was completely pulled into the sheath. This action was repeated three times with the last time removing the main bulk of the embolus. A control series showed complete removal of the foreign body with normal blood flow restored (Fig. 1c). A final picture of the leg arteries to prove absence of distal embolization of the foreign body with previously known chronic occlusion of the posterior tibial artery was done (Fig. 1d). The patient was postinterventional symptom free. A histological analysis of the removed material confirmed its origin as a foreign body (Fig. 1e).

## Discussion

Accidentally pushing the Mynx sealant into the artery during application of the MynxGrip closure device is an extremely rare complication which could result in a distal foreign body embolization with signs and symptoms of an acute limb ischemia. We believe that this complication is most probably due to a faulty application of the device where the PEG Sealant was pushed towards the external vessel wall without the intravascular balloon being sufficiently being pulled back to fully close the arteriotomy site from the inside. To avoid this, we suggest that during the application of the device it is critical to ensure complete withdrawal of the procedural sheath out of the vessel by opening the stopcock of the sheath after reaching the second resistance point (balloon pulled against the inner vessel wall) and making sure that there is absolutely no blood coming out from the sheath before proceeding with pushing the PEG Sealant towards the outer vessel wall (https://www.cardinalhealth.com/content/dam/corp/web/documents/brochure/CardinalHealth MynxGripVascularClosureDeviceProcedureGuide.pdf).

Based on previous experiences of removing dislocated coils using stent retrievers, we were able to implement the same technique in removing dislocated coils successfully to remove the Mynx sealant safely and effectively. In summary, endovascular removal of foreign bodies using stent retrievers can be considered an effective and safe treatment option.

## Conclusion

Accidentally pushing the Mynx sealant into the artery during application of the MynxGrip closure device is an extremely rare but still avoidable complication of this closure device. It could be simply avoided by making sure that the vascular sheath is completely removed from the vessel and that the intravascular balloon of the device is sufficiently pulled against the internal vessel wall before deployment of the PEG sealant. Using stent retrievers is an effective and safe method to recover a foreign body embolus and it is highly recommended to combine it with simultaneous aspiration using an aspiration catheter and successive aspiration from a closely placed vascular sheath for more efficiency.

## Data Availability

Not applicable.
